# Mindfulness facets differentially relate to self-reported negative and positive emotional eating types in treatment-seeking adults with overweight/obesity

**DOI:** 10.1007/s40519-023-01578-9

**Published:** 2023-06-23

**Authors:** Wesley R. Barnhart, Maria A. Kalantzis, Abby L. Braden

**Affiliations:** Department of Psychology, Bowling Green State University, Bowling Green, OH 43403 USA

**Keywords:** Disordered eating, Emotional eating, Mindfulness facets, Adults, Obesity

## Abstract

**Background:**

Mindfulness is a meaningful therapeutic target in the treatment of emotional eating in adults with overweight/obesity. Descriptive research mapping relations between mindfulness facets and emotional eating types in treatment-seeking adults with overweight/obesity is needed.

**Methods:**

Cross-sectional relations between mindfulness facets (i.e., acting with awareness, describe, non-judgment, non-reactive, and observe; Five Facet Mindfulness Questionnaire-Short Form) and emotional eating types (i.e., self-reported negative and positive emotional eating; Emotional Eating Scale-Revised, Emotional Appetite Questionnaire) were examined in a treatment-seeking sample of adults with overweight/obesity (*N* = 63).

**Results:**

Significant bivariate correlations revealed negative relations between mindfulness facets and emotional eating types. Multiple regressions revealed that higher describe (*β* = − 0.42, *p* = 0.004) mindfulness was associated with lower self-reported emotional eating–anger/anxiety; higher non-reactive (*β* = − 0.31, *p* = 0.01) and non-judgment (*β* = − 0.28, *p* = 0.02) mindfulness were associated with lower self-reported emotional eating-depression; and higher non-judgment (*β* = 0.26, *p* = 0.04) mindfulness was associated with higher self-reported emotional eating-positive.

**Conclusions:**

Describe, non-judgment, and non-reactive mindfulness were uniquely and significantly associated with eating in response to negative and positive emotions. Results suggest the potential need for intervention programs to be sensitive to the multidimensional nature of mindfulness in the treatment of distinct types of emotional eating in adults with overweight/obesity.

**Level of evidence:**

V, cross-sectional descriptive study.

People with overweight and obesity often report emotional eating, which is defined as the urge to eat in response to negative and positive emotional antecedents [1–3]. Emotional eating can be further defined (i.e., emotional eating types) as eating in response to depression (e.g., discouraged, loneliness, sadness), anger (e.g., furious, irritated), anxiety (e.g., worried), boredom (e.g., disinterest, unstimulated), and positive emotions (e.g., confident, happy, relaxed) [2–4]. Previous research suggests that upwards of 50–60% of adults with overweight/obesity report frequent engagement in emotional eating [5, 6]. Importantly, emotional eating is associated with poor physiological and psychological outcomes, including disordered eating [7, 8]. Indeed, some research suggests that negative emotional eating prospectively predicts disordered eating [9], making it an important treatment target in prevention research. Together, the common occurrence and negative biopsychological correlates of emotional eating underscore the importance of emotional eating research, especially among adults with overweight/obesity.

One theoretical model underlying emotional eating is maladaptive affect regulation. Maladaptive affect regulation includes difficulty regulating emotions and encompasses a range of difficulties around labeling, clarity, avoidance, and suppression of emotions [10, 11]. To date, maladaptive affect regulation has been used to explain emotional eating [1, 12], with findings suggesting that difficulty regulating negative emotions is positively associated with emotional eating. In addition to negative emotional eating, maladaptive affect regulation may also be associated with positive emotional eating. Emerging research suggests that maladaptive affect regulation can impact the experience of positive emotions, with difficulties observed in processing and making sense of positive emotional experiences (e.g., non-acceptance of positive emotions, difficulties controlling impulsive behavior and goal-directed behavior in the presence of positive emotions; [13, 14]). In the context of emotional eating, a recent study showed that perceived parental control of unhealthy eating behaviors and pressure to eat were more strongly associated with positive emotional eating in community adults reporting higher emotion regulation difficulties [15]. While future research is needed, maladaptive affect regulation may be a useful framework to explain negative and positive emotional eating. Indeed, this shared mechanism of maladaptive affect regulation reveals an important opportunity for research to explore therapeutic targets that may yield benefits across negative and positive emotional eating. One such therapeutic target is mindfulness.

Broadly, mindfulness includes a range of attitudes, cognitions, and behaviors that guide attention to the present moment [16]. More specifically, mindfulness includes attitudes of acceptance about one’s experiences in real-time, both positive and negative, as well as general beliefs of openness to experiences (e.g., increased openness and willingness to participate in new and potentially difficult experiences) [17]. Mindfulness also includes navigating one’s environment with awareness to the present moment. This includes being attentive to and not avoidant of one’s internal experience, including emotions and external experiences, such as physical surroundings [17, 18]. Research suggests that mindfulness processes exert their therapeutic effects by improving maladaptive affect regulation (e.g., correlational data [19]; experimental data [20]; review [21]). Thus, mindfulness may improve emotional eating by supporting an individual’s adaptive management of their emotions. In the context of treatment research, mindfulness-based interventions have decreased the severity of a range of maladaptive eating behaviors and improved health status among adults with overweight/obesity [22–26]. Historically, the literature has defined and validated self-report measures of mindfulness to be unidimensional (e.g., broad) or multidimensional (e.g., specific facets). The latter includes acting with awareness mindfulness, describe mindfulness, non-judgment mindfulness, non-reactive mindfulness, and observe mindfulness [27]. Briefly, acting with awareness mindfulness includes focused attention to the present moment, including one’s thoughts and emotions, as opposed to thinking about the past or future; describe mindfulness includes labeling experiences with language (e.g., “I’m noticing my muscles tensing up.”); non-reactive mindfulness includes letting thoughts and emotions be experienced without attentional engagement (e.g., not getting “stuck” on a thought or emotion and instead letting them naturally ebb and flow); non-judgment mindfulness includes acceptance and openness toward one’s internal state (e.g., responding neutrally instead of critically when experiencing a negative emotion); and observe mindfulness includes the active recognition of one’s external/sensory experiences (e.g., noticing taste, smell, textures, visual stimuli, sounds) [27]. Examining specific facets of mindfulness may provide detailed information about mindfulness’s therapeutic value across the spectrum of eating pathology.

To date, some research has explored mindfulness facets in relation to emotional eating. One recent meta-analysis [28] identified that emotional eating was strongly and inversely associated with mindfulness; however, in this particular study, emotional eating was combined with external eating (e.g., eating in response to external cues [sight and smell of food]). Thus, the unique relation between mindfulness facets and emotional eating, specifically negative and positive emotional eating and emotional eating types (e.g., eating in response to depression), is less clear. Importantly, this same meta-analysis identified that external/emotional eating, as opposed to general disordered eating, eating concerns (e.g., feeling guilty after eating), bulimic behavior, and dietary restraint, was more strongly associated with mindfulness which speaks to the importance of further investigating the relationship between these constructs [28]. One study identified that higher acting with awareness mindfulness, describe mindfulness, non-judgment mindfulness, and non-reactive mindfulness, but not observe mindfulness, were associated with lower negative emotional eating in a pre-surgical bariatric population [29]. In an online community sample with overweight weight status, higher acting with awareness mindfulness, describe mindfulness, and non-judgment mindfulness, but not non-reactive and observe mindfulness, were associated with lower negative emotional eating [30]. Interestingly, other research has suggested that more external/sensory-based facets of mindfulness such as observe mindfulness may be positively associated with negative emotional eating [30–32]. More specifically, paying greater attention to bodily sensations (e.g., item from the observe subscale of the Five Facet Mindfulness Questionnaire, “I notice how foods and drinks affect my thoughts, bodily sensations, and emotions.” [27]) and other sensory experiences (e.g., sights, sounds, and smells) may be triggers to maladaptive eating behaviors such as negative emotional eating. Consistent with that theory, using a longitudinal approach, one study identified that higher observe mindfulness assessed at baseline was predictive of worsened external eating and negative emotional eating 6 months later [32]. Taken together, findings with diverse populations suggest that at the facet level, acting with awareness mindfulness, non-judgment mindfulness, describe mindfulness, and non-reactive mindfulness appear to be consistently related to lower emotional eating. Thus, when people label their experiences in a non-judgmental way, maintain focus on the present, and notice experiences without immediately reacting, they may be less likely to engage in emotional eating. However, findings with observe mindfulness are mixed in relation to emotional eating, suggesting the need for continued research. Put another way, it is possible that paying close attention to bodily sensations and sensory experiences may be associated with less or more emotional eating. Importantly, no research has probed relations between mindfulness facets and specific types of self-reported negative emotional eating, such as eating in response to depression, anger/anxiety, and boredom, as well as its counterpart, positive emotional eating, in adults with overweight/obesity.

Examining associations between specific types of self-reported negative emotional eating and mindfulness facets is important given research suggests that negative emotional eating is a heterogenous construct [1, 33, 34]. Growing research suggests that some negative emotional eating types (e.g., eating in response to depression) may be more strongly associated with psychopathology (e.g., disordered eating) than other types of negative emotional eating (e.g., eating in response to boredom) [35, 36]. A recent study described frequencies and psychological correlates of such emotional eating types in a sample of treatment-seeking adults with overweight/obesity [36]. Findings suggested frequencies spanning 14.3–44.4% across emotional eating types, with eating in response to depression being the most frequently reported emotional eating type (44.4%) [36]. Furthermore, findings largely replicated previous research [35], suggesting that eating in response to depression was most closely associated with higher disordered eating, binge eating, and depressive symptoms compared to other emotional eating types [36]. Additional nuances from this study suggested that eating in response to anger/anxiety was most closely associated with emotion regulation difficulties compared to other emotional eating types [36]. It can therefore be argued that negative emotional eating types, as opposed to negative emotional eating more broadly, may be uniquely related to adaptive psychological processes such as mindfulness; however, no research to date has examined these speculations. Furthermore, no research to date has directly examined relationships between positive emotional eating and mindfulness facets, a concerning gap in the literature considering that negative and positive emotional eating types may occur at similar rates [37], that positive emotional eating is a common trigger for eating [38], and that positive emotional eating may be associated with negative psychological correlates (e.g., binge eating behavior; [39, 40]). As such, research examining relationships between mindfulness facets and positive emotional eating is needed to elucidate potential protective factors of this type of emotional eating.

## The present study

The current study explored relations between mindfulness facets and emotional eating in adults with overweight/obesity given that both mindfulness and emotional eating share theoretical mechanisms (e.g., maladaptive affect regulation [1, 12]). More specifically, we explored relations between acting with awareness mindfulness, describe mindfulness, non-reactive mindfulness, non-judgment mindfulness, and observe mindfulness facets and negative (including eating in response to anger/anxiety, boredom, and depression) and positive emotional eating types. Guided by previous research [28], exploratory hypotheses predicted that overall, negative relations would emerge between mindfulness facets and emotional eating types in adults with overweight/obesity. Specifically, we predicted that acting with awareness mindfulness, non-judgment mindfulness, describe mindfulness, and non-reactive mindfulness would be inversely associated with negative emotional eating types. To this end, the present study first examined bivariate relationships between mindfulness facets and types of emotional eating types (e.g., eating in response to depression, anger/anxiety, boredom, and positive emotional eating). Significant bivariate relations were further probed with multiple regressions to examine how much variance in emotional eating (e.g., eating in response to depression, anger/anxiety, boredom, and positive emotional eating) was explained by these mindfulness facets and whether any of the mindfulness facets were uniquely related to outcomes in a treatment-seeking sample of adults with overweight/obesity.

## Methods

### Participants and procedure

The present study received Institutional Review Board approval at a midwestern university. Community sampling was employed to recruit participants for a weight-loss program focused on emotion regulation skills. Specifically, participants were initially recruited via flyers, newspapers, social media, and emails to university students and faculty. Then, after initial phone screeners to determine eligibility, participants completed an orientation and baseline assessment for a pilot treatment program that focused on behavioral weight loss (BWL) and emotion regulation skills.

Inclusion criteria to be enrolled at baseline required a body mass index (BMI (kg/m2)) equal to or greater than 25 and equal to or greater than 22 years of age, no older than 65 years of age. The lower age limit was set to avoid recruiting a sample primarily consisting of traditional college students as the pilot treatment intervention was not tailored for this demographic. The upper age limit was set to be consistent with another pilot study that did not target weight loss but used DBT skills to target emotional eating [41]. Participants also had to have at least a 6th-grade reading level of the English language, able to attend the baseline and treatment sessions, and deny intent to move outside of their current residency. Exclusion criteria required that participants were not pregnant or currently breastfeeding; no engagement in purging or other compensatory behaviors in the last 3 months; no psychosis, substance misuse disorder(s), or severe depression symptoms with accompanying suicidality; no concurrent involvement in psychotherapy; no use of weight management medications; no changes in psychotropic medication in the last 3 months; and no current involvement in a weight loss intervention.

Participants completed a series of measures at baseline via Qualtrics, an online survey platform. Twenty-five participants at baseline did not complete or were ineligible for the pilot treatment program and 39 participants completed the treatment pilot program. The present sampling represents a secondary analysis of participants at baseline [42]. Demographic data were collected for a subset of participants. The final sample (*N* = 63) were adults with overweight/obesity (97% female) who self-identified as emotional eaters. Regarding emotional eater status, recruitment materials asked “Are you an emotional eater?” During the initial phone screening, participants were further asked what specific negative emotions trigger eating for them, as well as how often it happens. Participants had to report both negative emotional eating as well as report emotional eating as a maladaptive behavior. In addition, participants had to score above the means from a community sample of adults with overweight/obesity [35] on at least one subscale that assessed negative (i.e., EES-R) and positive (i.e., EMAQ) emotional eating. Participants were compensated with a 25-dollar gift card for their participation.

Participants were on average 49 years of age (*SD* = 10.9), predominately White non-Hispanic (95.2%), graduate degree holders (39.7%), earning an annual income of $75,000 or more (28.6%), employed full-time (47.6%), and married (44.4%). See Table 1 for more information.

**Table 1:** Sociodemographic characteristics of participants

	*n*	%
Gender (*n* = 63)		
Female	61	3.2
Male	2	96.8
*M* (*SD*) BMI (*n* = 39)	35.8 (6.84)	N/A
*M* (*SD*) age (*n* = 39)	49.2 (11.16)	N/A
Marital status (*n* = 43)		
Single	8	12.6
Married/partnered	30	46.9
Divorced/widowed	3	4.7
Other	1	1.6
Education level (*n* = 59)	22	44
High school or equivalent	2	3.1
Some college	10	15.6
Vocational/technical school	2	3.1
Bachelor’s degree	17	26.6
Graduate degree	28	43.8
Employment		
Unemployed	2	3.2
Student	2	3.2
Part-time	2	3.2
Full-time	30	47.6
Retired	3	4.7
Homemaker	3	4.7
Race identity (*n* = 43)	17	34
White Non-Latino	40	62.5
Black or African American	3	4.7
Annual household Income (*n* = 43)		
$24,999 or less	2	3.1
$25,000 to $49,999	8	12.5
$50,000 to $74,999	14	21.9
$75,000 or more	19	29.7

*M* mean; *SD* standard deviation

### Measures

#### Demographics

Participants self-reported demographic characteristics including age, gender, marital status, racial identity, education level, employment status, and level of income (see Table 1).

#### Emotional Appetite Questionnaire

The 5-item positive emotions subscale of the Emotional Appetite Questionnaire (EMAQ) [2, 3] was used to assess eating in response to positive emotions (e.g., happy) and situations (e.g., falling in love). Response options range from 1 to 9 and are anchored from “much less” to “much more.” Participants can also report “NA” if the item does not apply or “DK” if the participant does not know. In the present study, internal consistency reliability for the positive emotional eating subscale was acceptable (*ɑ* = 0.88).

#### Emotional Eating Scale-Revised

The Emotional Eating Scale-Revised (EES-R [4]) was developed from the Emotional Eating Scale (EES [1]) to include a boredom subscale. The EES-R is a Likert scale measurement assessing emotional eating across three subscales: anger/anxiety, boredom, and depression. A five-point Likert scale was used to anchor between “no desire to eat” to an “overwhelming desire to eat.” In the present study, internal consistency reliability for the depression, anger/anxiety, and boredom subscales were acceptable (*ɑ*s = 0.86, 0.79, and 0.82, respectively).

#### Five Facet Mindfulness Questionnaire-Short Form

The Five Facet Mindfulness Questionnaire-Short Form (FFMQ-SF) is a 24-item multifactorial scale that measures five facets of mindfulness (subscales): observe, describe, acting with awareness, non-judgment, and non-reactive [43]. Subscales, and not the total score, were used for analyses in the present study. A five-point Likert scale was used to anchor between “Never or very rarely true” to “Very often or always true.” In the present study, internal consistency reliabilities for the acting with awareness, describe, non-judgment, non-reactive, and observe mindfulness subscales were acceptable (*ɑ*s = 0.76, 0.86, 0.79, 0.82, and 0.77, respectively).

### Analytic plan

First, descriptive statistics and missingness of data were examined, followed with bivariate correlations across primary study variables (Table 2). Across bivariate correlational analyses, we examined relations between mindfulness facets (e.g., acting with awareness, describe, non-reactive, non-judgment, and observe) and emotional eating types (eating in response to depression, anxiety/anger, boredom, and positive emotional eating). Next, before calculating multiple regression analyses, assumptions of multiple regression, including homoscedasticity, linearity, and normality, were examined. Next, collinearity diagnostics were calculated to detect any threats of multicollinearity. Finally, significant bivariate relations were further probed with multiple regressions to examine how much variance in emotional eating was explained by these mindfulness facets and whether any of the mindfulness facets were uniquely related to outcomes. In total, three multiple regressions were calculated examining the variance contributed by mindfulness facets in relation to emotional eating–depression, emotional eating–anger/anxiety, and emotional eating-positive. Given that significant relationships were not observed between mindfulness facets and emotional eating-boredom, this emotional eating variable was not examined in regression analyses. See Table 3 for more information.

**Table 2:** Descriptive statistics and bivariate correlations among primary study variables

	*M*	*SD*	*Min.*	*Max.*	Range	Skew(*se*)	*1*. AA	*2*. DS	*3*. NR	*4*. NJ	*5*. OB	*6*. EE-AA	*7*. EE-B	*8*. EE-D
*1.* AA	2.99	0.72	1.2	4.6	3.4	0.05 (0.30)	–							
*2.* DS	3.39	0.75	2.0	5.0	3.0	0.29 (0.30)	0.58**	–						
*3*. NR	2.54	0.74	1.0	4.2	3.2	0.11 (0.30)	0.37**	0.21	–					
*4.* NJ	2.94	0.80	1.6	5.0	3.4	0.33 (0.30)	0.44**	0.19	0.20	–				
*5.* OB	3.39	0.81	1.25	5.0	3.75	− 0.57 (0.30)	0.30*	0.01	0.17	− 0.04	–			
*6*. EE-AA	3.18	0.79	1.25	4.63	3.38	− 0.40 (0.30)	− 0.29*	− 0.44**	− 0.28*	− 0.23	0.04	–		
*7*. EE-B	3.19	0.71	1.75	4.38	2.63	− 0.36 (0.30)	− 0.10	− 0.001	0.01	0.02	− 0.02	0.02	–	
*8*. EE-D	3.64	0.75	1.78	5.00	3.22	− 0.58 (0.30)	− 0.22	− 0.15	− 0.36**	− 0.34**	− 0.01	0.42**	0.35**	–
*9*. EE-P	4.50	1.32	1.40	7.27	5.87	− 0.17 (0.30)	0.13	0.03	0.04	0.27*	− 0.25*	0.06	0.07	− 0.01

*M* mean; *SD* standard deviation; *Min.* minimum; *Max.* maximum; *N* = 63

*AA* acting with awareness; *DS* describe; *NR* non-reactive; *NJ* non-judgment; *OB* observe; *EE–AA* emotional eating-anger/anxiety; *EE-B* emotional eating-boredom; *EE-D* emotional eating-depression; *EE-P* emotional eating-positive; *se* standard error

*p < 0.05; **p < 0.01

**Table 3:** Multiple regression analyses of mindfulness facets in relation to emotional eating

	*B*	SE_B_	*β*	*t*	*p*
Emotional eating-anger/anxiety					
Constant	5.13	0.49	–	10.5	< 0.001***
Acting with awareness	0.03	0.16	0.03	0.20	0.85
Describe	− 0.45	0.15	− 0.42	− 2.99	0.004**
Non-reactive	− 0.21	0.13	− 0.20	− 1.61	0.11
Emotional eating-depression					
Constant	5.20	0.41	–	12.8	< 0.001***
Non-reactive	− 0.31	0.12	− 0.31	− 2.61	0.01*
Non-judgment	− 0.26	0.11	− 0.28	− 2.36	0.02*
Emotional eating-positive					
Constant	4.68	0.92	–	5.08	< 0.001***
Non-judgment	0.42	0.20	0.26	2.14	0.04*
Observe	− 0.39	0.20	− 0.24	− 1.98	0.05

*p < 0.05; **p < 0.01; ***p < 0.001

## Results

### Preliminary results

See Table 1 for demographic characteristics of the current sample and Table 2 for bivariate correlations on constructs of interest. Across participants, missingness of data were low (0.02%; *n* = 1), resulting in the use of listwise deletion across primary analyses. Assumptions of multiple regression were confirmed via visual inspection of quantile–quantile (Q–Q) plots, scatterplots, and normality distributions. Furthermore, skewness across primary study variables were within acceptable ranges (e.g., ± 1; Table 2), suggesting that data across primary study variables were normally distributed. Collinearity diagnostics revealed no threats of multicollinearity as evidenced by tolerance and variance inflation factors within acceptable ranges.

### Bivariate relations between mindfulness facets and emotional eating types

Significant bivariate relations emerged between mindfulness facets and emotional eating (Table 2). Across emotional eating types, higher non-reactive (*r* = − 0.36, *p* < 0.01) and non-judgment (*r* = − 0.34, *p* < 0.01) mindfulness were associated with lower emotional eating–depression; higher non-reactive (*r* = − 0.28, *p* < 0.05), acting with awareness (*r* = − 0.29, *p* < 0.05), and describe (*r* = − 0.44, *p* < 0.01) mindfulness were associated with lower emotional eating–anger/anxiety; and higher observe (*r* = − 0.25, *p* < 0.05) and lower non-judgment (*r* = 0.27, *p* < 0.05) mindfulness were associated lower and higher emotional eating-positive, respectively.

### Multiple linear regressions

Three multiple linear regressions were examined with mindfulness facets entered as independent variables and emotional eating–anger/anxiety, emotional eating–depression, and emotional eating-positive as dependent variables (see below).

#### Emotional eating–anger/anxiety

A multiple linear regression was calculated to explain variance in emotional eating–anger/anxiety with the inclusion of non-reactive, acting with awareness, and describe mindfulness facets. A significant regression equation was found (*F*(3, 59) = 5.95, *p* = 0.001), with an *R*^*2*^ = 0.23 (Table 3). When examining specific mindfulness facets in the model, describe (*β* = − 0.42, *t* = − 2.99, *p* = 0.004) mindfulness was significantly associated with emotional eating–anger/anxiety such that higher describe mindfulness was associated with lower emotional eating–anger/anxiety. Non-reactive and acting with awareness mindfulness facets did not contribute significant variance in emotional eating–anger/anxiety (*ps* > 0.05).

#### Emotional eating–depression

A multiple linear regression was calculated to explain variance in emotional eating–depression with the inclusion of non-reactive and non-judgment mindfulness facets. A significant regression equation was found (*F*(2, 60) = 7.23, *p* = 0.001), with an *R*^*2*^ = 0.21 (Table 3). When examining specific mindfulness facets in the model, both non-reactive (*β* = − 0.31, *t* = − 2.61, *p* = 0.01) and non-judgment (*β* = − 0.28, *t* = − 2.36, *p* = 0.02) mindfulness were significantly associated with emotional eating–depression such that higher non-reactive and non-judgment mindfulness were associated with lower emotional eating–depression.

#### Emotional eating-positive

A multiple linear regression was calculated to explain variance in emotional eating-positive with the inclusion of observe and non-judgment mindfulness facets. The regression equation was significant (*F*(2, 60) = 4.44, *p* = 0.02), with an *R*^2^ = 0.13 (Table 3). When examining specific mindfulness facets in the model, non-judgment (*β* = 0.26, *t* = 2.14, *p* = 0.04) mindfulness was significantly associated with emotional eating-positive such that higher non-judgment mindfulness was associated with higher emotional eating-positive. Observe (*β* = − 0.24, *t* = − 1.98, *p* = 0.05) mindfulness approached significance such that higher observe mindfulness was associated with lower emotional eating-positive; however, this relationship was ultimately not significant (*p* ≥ 0.05).

## Discussion

Given no research to date has examined relationships between mindfulness facets and negative emotional eating types (eating in response to depression, anger/anxiety, boredom) and positive emotional eating, the present study examined relations between mindfulness facets and these self-reported emotional eating types in a treatment-seeking sample of adults with overweight/obesity and emotional eating. Unique mindfulness facets were related to eating in response to depression, anger/anxiety, and positive emotions. At the bivariate level, null relations were observed between mindfulness facets and eating in response to boredom which may speak to the limited utility of mindfulness in the treatment of boredom-induced maladaptive eating behaviors. Such findings may speak the more innocuous nature of boredom affective experiences compared to depression and anger/anxiety; however, future research should replicate this finding given previous research suggesting that boredom is a significantly distressing affective experience associated with poor psychological and physical health outcomes [44]. Patterns of relationships between mindfulness facets and emotional eating showed that acting with awareness, non-reactive, and non-judgment mindfulness comprised most of the significant negative relations.

Regression results showed that non-reactive and non-judgment mindfulness were uniquely associated with emotional eating–depression. Like above, these findings may be informed through maladaptive affect regulation theory: open and accepting reactions (i.e., non-judgment mindfulness) to negative emotional experiences, as well as a tendency to let negative emotional experiences come and go without attentional intervention (i.e., non-reactive mindfulness), may be closely related to self-reported eating in response to depression. For some, it may be that self-judgement in response to negative emotions (e.g., “I hate myself for being sad.”) engages emotional eating to cope with these difficult affective experiences; thus, approaching emotional antecedents with non-judgment (e.g., “Sadness can happen from time to time.”) may weaken the need for maladaptive eating to cope with these difficult affective experiences. Non-reactive mindfulness may work in a similar way by simply reflecting a tendency to not engage with such negative affective experiences, thereby rendering sadness, loneliness, and discouragement as less potent triggers of emotional eating. Findings are in line with research that has shown negative relations between non-judgment and non-reactive mindfulness and broad, self-reported negative emotional eating in a pre-surgical bariatric population [29]. These findings with adults with overweight/obesity were also consistent with previous research that showed negative relationships between non-judgment and non-reactive mindfulness and emotional eating–depression in an online community sample [30]. Importantly, the above speculations imply temporal order and future longitudinal research is needed to validate these relations.

Like emotional eating–depression, mindfulness facets were also uniquely associated with self-reported emotional eating–anger/anxiety. More specifically, describe mindfulness was uniquely, negatively associated with emotional eating–anxiety/anger in the regression model. Though speculative in nature, describe mindfulness, which includes labeling experiences with language, may be negatively associated with eating triggered by anxiety by bringing such negative emotions to one’s active awareness. Put another way, the use of language to label emotions such as feeling furious, irritated, and worried (e.g., “I am feeling worried, angry, and annoyed right now.”) may help bring insight to one’s internal state and weaken the need for emotional eating to cope with these difficult affective experiences. In this way, the inverse relation between describe mindfulness and emotional eating–anger/anxiety may also be explained through maladaptive affect regulation: the act of not labeling one’s experiences (i.e., negative emotional experiences) with language may support avoidance or suppression of these emotions which, therefore, may make them more viable triggers of non-homeostatic eating behaviors. These findings overlap with emotion regulation research suggesting that difficulties labeling emotions may contribute to negative psychological outcomes including maladaptive eating behaviors (e.g., alexithymia; disordered eating [45], negative emotional eating [46]). Furthermore, these findings add to existing research suggesting negative (e.g., broad negative emotional eating [29]; emotional eating–depression [30]) relations between describe mindfulness and emotional eating. It may be the case that describe mindfulness, including using language to describe emotions [47], is particularly useful for negative emotions, such as anger and anxiety. Importantly, continued research is needed to better understand what transpires after individuals label their affective experiences in relation to emotional eating. It may be the case that labelling affective experiences is useful only in circumstances where other, healthy coping strategies are in place (e.g., deep breathing, cognitive restructuring). These findings with self-reported emotional eating–depression and emotional eating–anger/anxiety should be considered alongside those with emotional eating-positive.

The regression model explaining variance in emotional eating-positive showed that higher observe mindfulness was trending (*β* = − 0.24, *p* = 0.05) toward lower self-reported emotional eating-positive; however, this relationship was ultimately not significant. That said, higher non-judgment mindfulness was uniquely and significantly associated with *higher* self-reported emotional eating-positive. Considering emotional eating-positive within the maladaptive affect regulation framework, it may be the case that difficulties regulating negative emotions may be related to a greater up-regulation of positive emotions. To this end, observe mindfulness, which focuses on bringing attention to one’s sensory experiences, may de-emphasize internal experiences of positive emotions and thereby make such triggers less viable in the context of eating behavior among adults with overweight/obesity. Importantly, findings on the inverse relation between observe mindfulness and emotional eating-positive diverge from previous studies [30–32, 48, 49] suggesting that observe mindfulness is a *positive correlate* of eating pathology. These findings may speak to growing research suggesting that emotional eating-positive is distinct from its negative counterpart [35, 36] and related constructs, such as disordered eating. Still, caution should be taken with interpreting these findings given this relationship was trending toward but ultimately not significant. Findings on the positive relation between non-judgment mindfulness and emotional eating-positive also contribute to this research and outline the continued need for future research to distinguish negative and positive emotional eating.

The present findings have various clinical implications. With future replication efforts using longitudinal designs to confirm the temporal order of study variables, these preliminary findings support focusing on describe (e.g., using language to label emotions) mindfulness in the treatment of eating in response to anger and anxiety and non-reactive and non-judgment mindfulness in the treatment of eating in response to depression. Broadly, findings also have implications for the operationalization of mindfulness and emotional eating: unique and differential relationships were observed between mindfulness facets and self-reported emotional eating types, findings that may provide a finer level of detail relevant to researchers and clinicians alike. While the present findings represent a useful addition to the literature, they are not without limitations and future directions.

### Limitations and future directions

First, the present sample of treatment-seeking adults with overweight/obesity was small and homogenous (e.g., 97% female; 92.5% White). Future research should seek to replicate these findings in a larger, more heterogeneous sample of adults with overweight/obesity, particularly with the inclusion of racially diverse women, men, non-binary, and sexual minority (e.g., LGBTQ+) participants to provide more comprehensive and inclusive conclusions about the observed relations between mindfulness facets and emotional eating types. Furthermore, other participant characteristics (e.g., BMI, income level, treatment-seeking status) of the sampled population were homogenous and may have influenced the present findings; thus, future research is needed to replicate and expand on these presented relationships in non-clinical participants at various levels of BMI, weight status, and across the lifespan. Second, the present study did not exclude participants with binge eating disorder or atypical anorexia nervosa. Relatedly, findings should only be generalized to adults with overweight/obesity and not eating disorder populations (e.g., binge eating disorder) or people without overweight/obesity. Third, the present study used self-report measures to probe emotional eating. Growing research underscores limited overlap between self-report measures, experience sampling, and experimental assessments of emotional eating [50, 51]. To this end, findings from the present study should only be generalized to self-reported emotional eating and future research efforts should aim to replicate the observed relations with diverse types of emotional eating assessment. Fourth, the present study did not include a self-report measure of positive emotional eating types (i.e., positive emotional eating was measured as a unidimensional construct), a limitation that represents an important future research direction in the service of expanding these findings. Finally, because the present study was cross-sectional in nature, no information about the causal or temporal order of study variables can be discerned.

## Conclusions

Research underscores the utility of mindfulness to reduce the severity of emotional eating in populations with elevated weight status. The present findings contribute to this research by describing differential relations between mindfulness facets and emotional eating types in a treatment-seeking sample of adults with overweight/obesity. Specifically, non-reactive, describe, observe, and non-judgment mindfulness facets were uniquely associated with self-reported emotional eating across negative and positive dimensions among adults with overweight/obesity. With replication, findings suggest the potential need for intervention programs to be sensitive to the multidimensional nature of mindfulness in the treatment of distinct types of emotional eating in adults with overweight/obesity.

## Strengths and limits

The key strengths of this study include that it examines associations between specific emotional eating types (e.g., eating in response to depression, anger/anxiety, boredom, and positive emotions) and mindfulness facets in a clinical, treatment-seeking sample. This is important because emotional eating types are uniquely related to psychological health outcomes. These findings, of which no research to date has directly tested, add a finer level of detail on the nature of associations between mindfulness facets and emotional eating types. The key limits of this study include its cross-sectional design and small, homogenous sample size.

## Data Availability

The data sets generated during and/or analysed during the current study are available from the corresponding author on reasonable request.
